# The senescence-associated secretory phenotype induces neuroendocrine transdifferentiation

**DOI:** 10.18632/aging.204669

**Published:** 2023-04-17

**Authors:** Anda Huna, Nadine Martin, David Bernard

**Affiliations:** 1 Centre de Recherche en Cancérologie de Lyon, Inserm U1052, CNRS UMR 5286, Centre Léon Bérard, Université de Lyon, Equipe Labellisée la Ligue Contre le Cancer, France

**Keywords:** cellular senescence, aging, cancer, NF-κB, calcium signaling

The senescence-associated secretory phenotype (SASP), in addition to stable proliferation arrest, is one of the most remarkable characteristics of senescent cells. Indeed, these cells secrete a variety of factors including cytokines, growth factors and matrix metalloproteases among others. In response to stress, through their SASP, senescent cells are able to modify and instruct their microenvironment. The SASP is known to have several, sometimes contradictory, effects on phenotypes, including the induction or reinforcement of senescence in neighboring cells, promotion or inhibition of stemness, modification of extracellular matrix, activation or inhibition of immune responses and induction of epithelial-mesenchymal transition and cell migration. Although cellular senescence and its SASP can initially display some beneficial effects, for instance favoring wound healing or blocking tumor initiation, accumulation of senescent cells and their secretome during aging or chronic stresses (tobacco, obesity, alcohol among others) plays a significant role in promoting aging-associated features and pathologies, like fibrosis, steatosis, chronic inflammation or cancer [1].

In the context of cancer, senescence initially has an anti-tumoral role, as it promotes proliferation arrest and favors an anti-tumoral immune surveillance in response to oncogenic stress or DNA damage accumulation. However, SASP plays a dual role in tumor initiation and progression, as it first has a tumor suppressive action by reinforcing senescence in neighboring cells and recruiting immune cells, but also plays a tumor promoting role by promoting stemness, epithelial-mesenchymal transition and cell migration and by inhibiting immune responses [2].

We recently unveiled a new interesting role for SASP: its ability to induce neuroendocrine transdifferentiation (NED) in breast cancer epithelial cells [3]. NED in tumors has been well described in prostate where it is thought to contribute to tumor resistance. In prostate cancer NED is characterized by the presence of neuroendocrine (NE) cell foci among the malignant cells. Androgen deprivation, used in therapy against hormone-dependent prostate cancer, has been described as one of the main inducers of NED, which is associated with a worse prognosis [4]. In the context of breast cancer, neuroendocrine tumors account for a small proportion (2–5%) of all tumors. They are poorly understood and whether classical breast tumors (of epithelial origin) can transdifferentiate, as do prostate tumors, is unclear [5].

**Figure 1:**
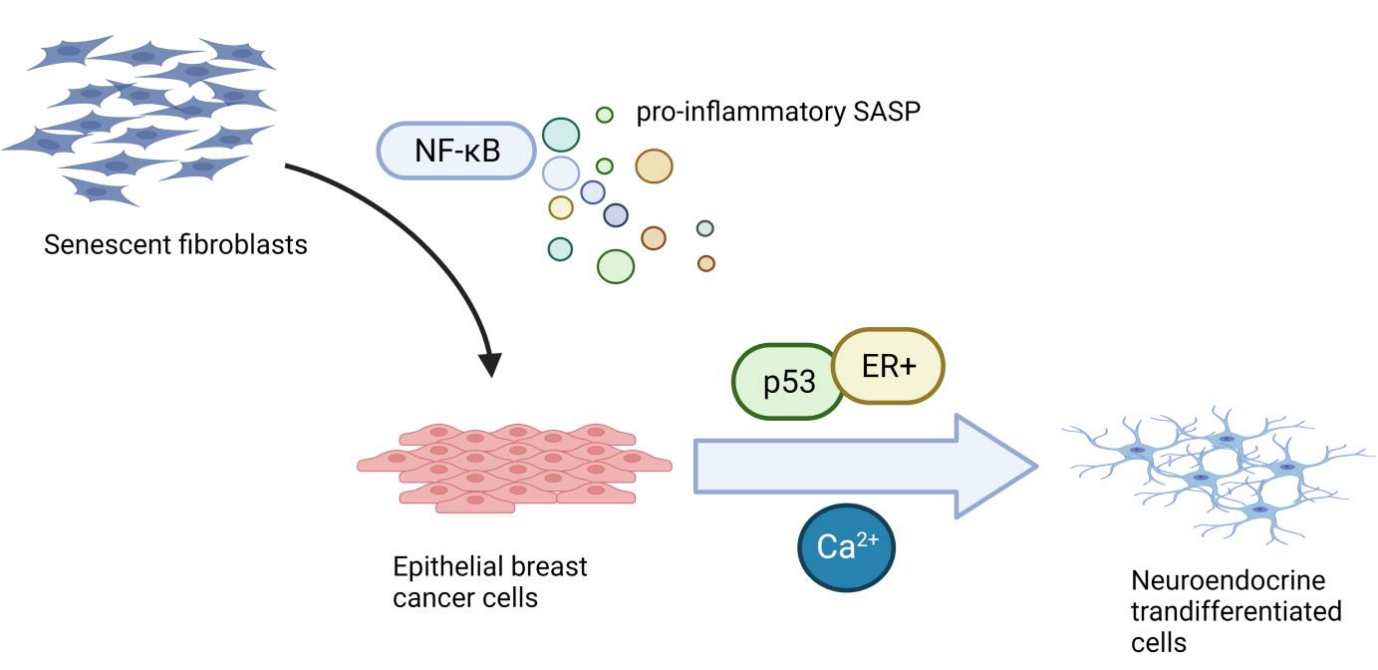
**Cell culture supernatant, containing NF-κB-dependent pro-inflammatory senescence-associated secretome (SASP), was collected from senescent normal human fibroblasts and used to treat human epithelial breast cancer cells.** It induced senescence and neuroendocrine transdifferentiation (NED), the latter being regulated by p53 and calcium in ER+ cells.

To examine the effects of SASP on breast cancer cells we collected conditioned medium from senescent human fibroblasts, a component of the tumor stroma. Different breast cancer cell lines were then treated with this SASP factor-containing medium or with conditioned medium from non-senescent control cells. In TP53-mutated (T47D and MDA-MB231) and/or ER-negative (MDA-MD231) breast cancer cells, we detected no significant differences in cells treated with SASP, however in MCF7 cells, which are TP53 WT and ER-positive, we observed marks of cellular senescence. Surprisingly, we also observed the unexpected appearance of ramifications reminiscent of neuroendocrine cells and an increased expression of neuroendocrine markers SCG2 and CHGB in these cells, indicating that SASP is able to induce NED in breast cancer epithelial cells. Interestingly, TP53 knockout partially impaired NED, but did not affect cell senescence induction, supporting that the NED process requires functional TP53. Similarly, SASP induced NED in TP53 WT LNCaP prostate cancer cells, but not in dysfunctional TP53 and AR-negative DU145 and PC-3 prostate cancer cells.

SASP is regulated by several signaling pathways including NF-κB, mTOR, C/EBPβ, p38MAPK among others [1]. NF-κB is a key regulator of inflammatory SASP [6]. By inhibiting NF-κB, and thereby decreasing levels of pro-inflammatory SASP members, NED was partially reverted.

Little is known on the molecular regulation of NED. It has been described that calcium signaling may be involved [7]. We detected increased calcium in senescent NED cells, mainly in intracellular stocks. Moreover, by buffering calcium, neurite-like structures and neuroendocrine gene expression were partially reverted.

To further understand the biology of NE breast tumors, we used Molecular Taxonomy of Breast Cancer International Consortium (METABRIC) database and isolated tumors which expressed neuroendocrine markers SCG2, CHGB, CHGA and SYP. This resulted in a group comprising 45 tumors of the 1,904 tumors included in the database, corresponding to the proportion of 2–5% of neuroendocrine tumors described for the breast. We performed Gene Set Enrichment Analysis (GSEA) based on the changes in gene expression between NE tumors and non-NE tumors, defined as negative for all four markers, and confirmed the enrichment of molecular signatures linked to NED such as neurotransmitter transport or signal release GO signatures. Furthermore, we observed upregulation in the GO signature of calcium signaling. All of these tumors were ER positive and predominantly TP53 WT, matching our experimental data. The proportion of NE breast tumors increased with age, which could be explained by senescent cell accumulation that could, over time, promote NED.

In our study, we identify a new role for SASP in inducing NED in some breast epithelial cells. Mechanistically this phenotype relies on NF-κB-dependent SASP and induction of Ca^2+^ signaling (Figure 1). It yet remains to be determined which inflammatory SASP factors play a role in NED induction.

Additionally, we observed increased calcium signaling in senescent or NED cells. It is well known that calcium signaling is essential to cell senescence, and an increase in calcium is observed in senescent cells [8]. However, interplay between calcium signaling, SASP and NED remains unclear, meaning that ion channels involved and precise calcium-activated downstream pathways have yet to be identified.

These observations in cell lines may carry clinical relevance, as they were validated by the analysis of the METABRIC database. To our knowledge, this is the first time such an approach is applied to the characterization of NE breast carcinomas. Consistent with our cell culture experiments we found a connection between the ER+ phenotype, p53 and calcium signaling and neuroendocrine markers.

Overall our work reveals a new effect of senescent cells and their SASP in tumors and offers new insights into NED in breast and prostate cancer biology. It also provides a new vision of the contribution of senescent cells and their SASP to aging-related pathologies, which could involve NED induction in some contexts.
